# Sustainable development education, practice, and research: an indigenous model of sustainable development at the College of Menominee Nation, Keshena, WI, USA

**DOI:** 10.1007/s11625-015-0304-x

**Published:** 2015-04-25

**Authors:** Michael J. Dockry, Katherine Hall, William Van Lopik, Christopher M. Caldwell

**Affiliations:** 1 US Forest Service, Northern Research Station, 1992 Folwell Ave., Saint Paul, MN 55108 USA; 2 College of Menominee Nation, N172 Wisconsin State Highway 47/55, Keshena, WI 54135 USA; 3 College of Menominee Nation, Sustainable Development Institute, N172 Wisconsin State Highway 47/55, Keshena, WI 54135 USA

**Keywords:** Indigenous wisdom, Sustainability models, Sustainability education, Menominee, Tribal Colleges and Universities

## Abstract

The College of Menominee Nation Sustainable Development Institute’s theoretical model (SDI model) conceptualizes sustainable development as the process of maintaining the balance and reconciling the inherent tensions among six dimensions of sustainability: land and sovereignty; natural environment (including human beings); institutions; technology; economy; and human perception, activity, and behavior. Each dimension is understood to be dynamic, both internally and in relationship to each of the other five dimensions. Change within one dimension will impact other dimensions in a continual process of change. Change can be externally driven or inherent to the dynamic nature of any of the six dimensions. Sustainable development is a continual and iterative process. A central concept of the model is based on the experience of the Menominee Indian Tribe of Wisconsin and their profound sense of place and relationship with the land that has allowed their community to recognize and balance the tensions among model dimensions through time. This paper provides a detailed description of the SDI model and its development and concludes with short examples illustrating how the model has been used for course design and delivery in higher education, interdisciplinary community planning, and participatory research.

## Introduction

Sustainability science is fundamentally interested in interdisciplinary approaches, integrative analysis, and practical applications linking knowledge to action in order to solve sustainability challenges (Kauffman 2009). Sustainability science provides practical applications for research into coupled human and environment systems (Miller 2013). One of the challenges of scientists and practitioners using sustainability science is to develop novel “integrated place-based models that are based on semi-qualitative representations of entire classes of dynamic behavior” (Kates et al. 2001). The Sustainable Development Institute (SDI) at the College of Menominee Nation (CMN)—an indigenous, tribally chartered, land-grant college located in Keshena, Wisconsin, USA—has developed just such an integrative and practical model of sustainable development based upon the Menominee Nation’s long-term experiences and understandings of sustainability. While based upon the Menominee experience, the SDI model can be used to understand universal principles of sustainability and can be an effective model to integrate sustainability science into interdisciplinary projects for both American Indian and non-Indian communities. Communities, planners, development workers, academics, and anyone striving to understand sustainability can use the SDI model to develop dynamic semi-qualitative narratives that can define current environmental problems, craft solutions, and develop visions for the future. This paper provides a description of the SDI model and its development and concludes with short examples illustrating how the SDI model has been used for course design and delivery in higher education, interdisciplinary community planning, and participatory research.

Models influence how researchers perceive and understand complex social and environmental systems (Taylor 2005). Because of this, researchers can develop different interpretations of the same coupled human and environment systems depending on the type of sustainability model used. Typical sustainability models have focused on three broad categories—environment, society, and economics. These tripartite models include the triple bottom line used extensively by the business community or the National Environmental Policy Act analysis used by the United States government (National Environmental Policy Act 1969; Elkington 1994). Tripartite models of sustainability have moved sustainability science forward over the last four decades; however, they often reduce complex human–environment interactions, culture, history, and institutions into a category called “social” (Giddings et al. 2002). The simplification of integrated systems also makes analysis of the interactions among the three categories difficult. These models are especially problematic for indigenous communities who often understand human and natural communities as integrated wholes (Jostad et al. 1996; YoungBear-Tibbetts et al. 2005; Berkes 2012).

Many indigenous communities are searching for meaningful and culturally appropriate ways to understand, measure, teach, and practice sustainable development and sustainable natural resource management (McGregor 2004; Corntassel 2008; Whyte 2014). Sustainability models used throughout the world can be problematic for indigenous communities because they do not often address or incorporate indigenous cultural values, concerns, world views (epistemologies and ontologies) or teachings. Indigenous concepts often excluded from sustainability models include reciprocity (mutual responsibilities guiding human and non-human interactions), interrelationships among humans and non-humans (all things are related), cooperation, and respect (Pierotti and Wildcat 2000; Salmon 2000; McGregor 2005; Ratner and Holen 2007; Kimmerer 2013). Another value shared by many indigenous communities, including the Menominee, is that the health of the land and people are one; a healthy forest means a healthy human community and a healthy human community means a healthy forest (Pecore 1992; Berkes 2012). Finally, sustainability models that use three general categories—economic, social, and environmental—may fail to account for dynamic interactions among categories because they tend to overlook history, changes through time, and often do not explore possible futures. They may also fail to incorporate complexity at multiple scales and the complexities of culture.

In this paper, we outline a model developed by the College of Menominee Nation’s Sustainable Development Institute in Keshena, Wisconsin. The SDI model was designed to allow for dynamic interactions among the model elements. It can incorporate history, change, possible futures, complexity at multiple scales, and culture. It can guide planners, foresters, educators, and community members in constructing dynamic narrative models to understand sustainability, make decisions, design research, and plan for the future. This paper concludes with examples of how the SDI model has been used to provide a more integrative perspective of sustainability in education, community planning and participatory research.

## Menominee sustainability and the origins of the Sustainable Development Institute model

The Menominee Nation has lived and managed their natural resources in the area of Northeast Wisconsin for thousands of years (Grignon et al. 2004). The Menominee Nation has managed their forests for sustainable timber supplies since 1856 when their current reservation was established (Pecore 1992; Hosmer 1999; Beck 2005). Some of the first federal laws mandating sustainable forest harvesting in the United States were enacted on the Menominee Indian Reservation marking the birth of sustainable forestry in the United States (Beck 2005; Dockry 2012). Today, Menominee Tribal Enterprises oversees tribal forestry and sawmill operations through a board of directors comprised of and elected by Menominee Nation tribal members. Menominee forestry continues to be world renowned for producing high-quality timber and economic resources for their community while maintaining and enhancing the health of their forest ecosystems (Pecore 1992).

The College of Menominee Nation is one of thirty-eight Tribal Colleges and Universities (TCUs) in the United States and Canada (see the American Indian Higher Education Consortium at http://www.aihec.org for information about TCUs). Tribal Colleges and Universities were founded by American Indian tribes starting in the late 1960s to provide culturally supportive environments for American Indian students, support local tribal communities, and produce indigenous research and scholarship (Boyer 1997). CMN was chartered by the Menominee Tribal Legislature in 1993 and reaffirmed by a vote from the general membership of the Menominee Tribe in 1996. CMN’s mission is “to provide opportunities in higher education to its students. As an institution of higher learning chartered by the Menominee People, the College infuses this education with American Indian culture, preparing students for leadership, careers and advanced studies in a multicultural world” (CMN 2013). From CMN’s founding there has been a strong connection and commitment to sustainability. Dr. Verna Fowler, founding CMN president, describes the deep connection between sustainability and education in an open letter stating that, “for our College and the Menominee People who chartered CMN, sustainable development has roots in the moral code, governance structure, and sustainable forestry practices that evolved within the tribe over many centuries. Since its beginning in 1993, the College of Menominee Nation has built its curriculum around these concepts and values. At the core are respect for the land, water, and air; partnership with other creatures of earth; and a way of living and working that achieves a balance between use and replenishment of all resources” (Fowler 2013). Sustainability has always been a part of Menominee life, economy, and education and it is infused throughout CMN.

In 1993, the Menominee Tribe initiated a joint project developed by CMN and Menominee Tribal Enterprises that facilitated the creation of SDI. The initial SDI mission was to promote the Menominee forest and its management through public education, focused on Menominee youth, and to facilitate forest-based economic development (CMN 1994). The initial development of SDI was guided by a Board of Directors made up of tribal leaders, CMN, the Menominee Tribal Legislature, and Menominee Tribal Enterprises. A representative from the Bureau of Indian Affairs was also included to reflect the trust responsibility the federal government has for the Menominee forest and tribe.

Throughout 1994, the Board of Directors developed a mission statement for SDI which stated, “To continuously expand knowledge, understanding and resources related to Menominee Nation Sustainable Development for the purpose of ensuring ongoing protection, control and productivity of the Menominee culture, environment, economy, technology, and community” (CMN 1994). To advance this mission, the SDI Board of Directors began to develop a theoretical model of sustainable development to understand the success of Menominee forest management, to share the sustainability successes with others, and to begin to address sustainability issues in other aspects of tribal life. SDI began with a framework of sustainable development outlined in both the CMN and SDI mission statements which postulated that sustainability comprises: community, technology, culture, governance, interconnectedness, economy, and tribal control. These sustainability elements were similar to work being done by the Forestry for Sustainable Development Program at the University of Minnesota at the time (see http://www.forestry.umn.edu/Publications/FFSD/) that identified five components of sustainable development: environmental, community, institutional, economic, and technology. In 1995, SDI created a Sustainable Development Advisory Council that paired Menominee leaders and tribal experts with external partners and experts loosely grouped around each of the five components of the initial model (Table 1). The Advisory Council built upon the CMN and SDI mission statements and the Forestry for Sustainable Development Program to develop a theoretical model of sustainable development to guide research, education, and outreach.

**Table 1:** 1995 Menominee Advisory Council on Sustainable Development

Early SDI model dimensions	Menominee leaders/experts	External partners/experts
Environmental	Forest Manager—Menominee Tribal Enterprises	Dean of the College of Natural Resources—University of Wisconsin Stevens Point
Community	Menominee Indian Tribe of Wisconsin, Tribal Historic Preservation Office	Professor of Sociology—University of Wisconsin Madison
Institutional	Former Tribal Chairman of Menominee Indian Tribe of Wisconsin	Professor of Political and Environmental Studies—University of Wisconsin Green Bay
Economic	Director of Menominee Economic Development	Director of the Land Tenure Center—University of Wisconsin Madison
Technology	President—Menominee Tribal Enterprises	Principle Engineer, Mater Engineering LTD

The Advisory Council comprised Menominee leaders/experts and external partners/experts. The advisory council was loosely based upon an early version of the SDI model. The overall process was convened by the President of the College of Menominee Nation and the Director of the Sustainable Development Institute

Over the next several years CMN, SDI, and the Sustainable Development Advisory Council convened meetings and held discussions among themselves, Menominee Tribal leaders, academics, and community members to understand the Menominee sustainable development experience and to build the SDI model. There is some debate within SDI and the Menominee community as to how much general community input went into the creation of the SDI model. Records pertaining to the development of the SDI model are not organized yet into CMN’s archives making it difficult if not impossible to understand the level of general community input into the process. What is clear from SDI’s records is that over time there were several SDI directors who each guided the process and brought their own perspectives into the dialogue. For example, during the mid-2000s, SDI embarked upon a process to reflect upon the SDI model, involve community members, and discuss Menominee cultural values and their relationship with sustainability. In the end, the SDI model developed over many years by expanding upon and integrating the Menominee experience and the CMN and SDI missions.

## The Sustainable Development Institute model

**Fig. 1:**
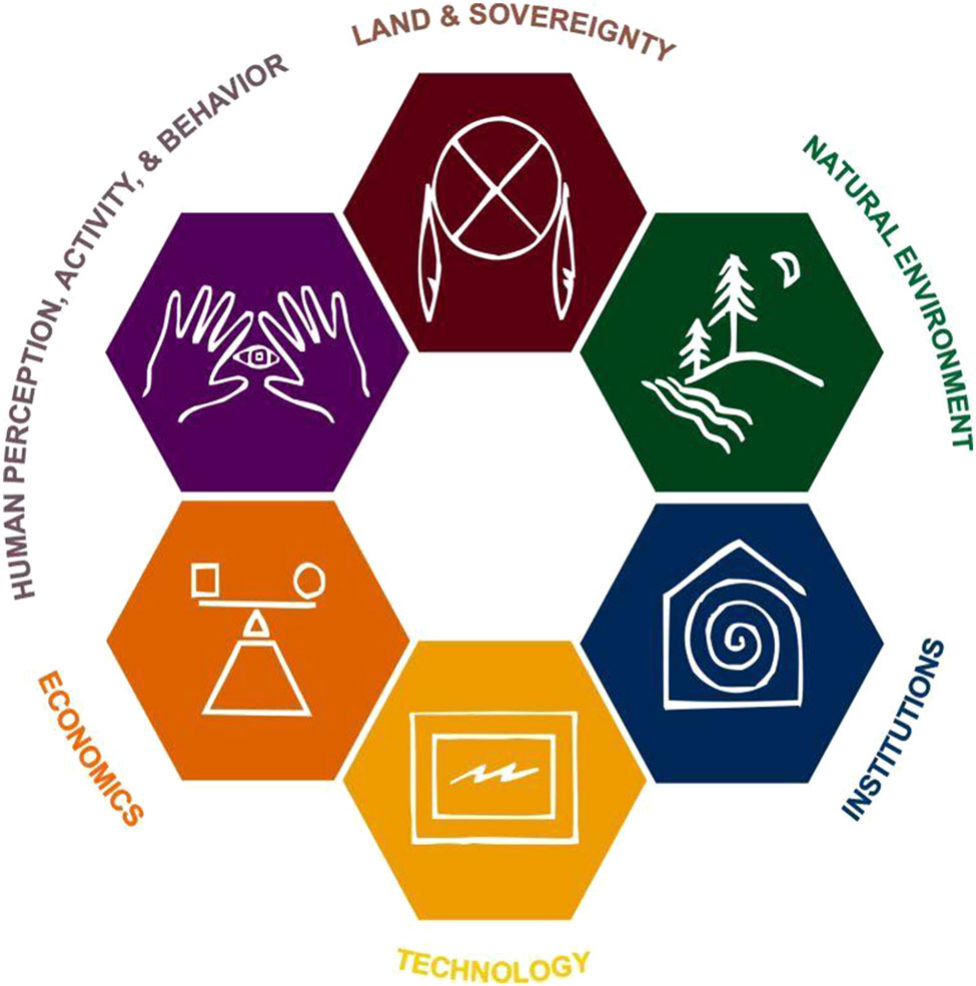
Six dimensions of sustainable development in the College of Menominee Nation Sustainable Development Institute’s Model. Menominee Autochthony (their profound sense of place and tie to the land) would occupy the center of the model and represent the Menominee cultural value that has allowed them to balance the tensions among the six model dimensions. Other communities using the model can identify their own cultural values that would allow them to balance the tensions among model elements (CMN c. 1999)

What emerged from the CMN and SDI process in the mid-1990s was a model that defines sustainability as comprising six discrete but highly interrelated dimensions: (1) land and sovereignty; (2) natural environment (which includes human beings); (3) institutions; (4) technology; (5) economics; and (6) human perception, activity, and behavior (Fig. 1). Land and sovereignty has specific legal and cultural meanings for the Menominee and other American Indian people that reminds us that they were, and continue to be, in sovereign control over their territories long before the United States government was formed (see Deloria and Lytle 1984). For non-American Indian communities, the land and sovereignty dimension is concerned with how decisions are made for their land and community. This dimension is important to the Menominee tribe because they have fought to retain their land and sovereignty for centuries (Beck 2002, 2005; Grignon et al. 2004; Peroff 2006). Menominee view this long struggle as one of the reasons they have been able to maintain a reservation within their ancestral territory, maintain the ecological diversity of their forestland through time, develop a world renowned forest management system, and establish CMN. The natural environment dimension of the SDI model is broadly interpreted to go beyond natural resources to include examples such as people, human communities, plants, animals, rocks, water, and air. The natural environment dimension incorporates Menominee understandings that everything is connected and related. The natural environment dimension can also incorporate western ecological science perspectives. Institutions in the SDI model refers to structures that develop and enforce rules of behavior and social interactions (which can include interactions among humans, plants and animals, and the environment) (see Ostrom 1986; Hodgson 2006 for multiple definitions of institutions). For the Menominee, institutions include things like the Menominee clan system, the modern tribal government, and CMN. Technology in the SDI model initially focused on rural community access to modern advances in telecommunications but later expanded to include cultural tools and practices. It includes Menominee technology for building birch bark canoes, processing wild rice, producing high-quality saw timber in a modern sawmill, and using Geographic Information Systems to implement sustainable forestry management activities. Today, technology can be understood as “how humans do things…or how humans get things done” (Dator et al. 2015). Economics is an important dimension found in many models of sustainability. For the SDI model, economics incorporates multiple scales ranging from the individual household, to the tribe, to the region, to the nation, to the globe. Economics for the Menominee includes the coexistence of individuals engaged in subsistence harvesting and commercial timber harvesting for sale onto the international market. The final SDI model dimension is human perception, activity, and behavior. This dimension includes different scales ranging from individual perceptions, activities and behaviors to community understandings, values, and collective pursuits. This dimension incorporates everything from Menominee cultural beliefs and practices to the creation of forestry management plans that limit timber harvesting to sustainable levels.

According to the SDI model, sustainable development is defined as the process of maintaining balance and reconciling the tensions within and among the six dimensions of sustainability. This does not mean to imply that there is a functional equilibrium or a “natural” balance; change is an explicit feature of the model. Each SDI model dimension is dynamic, both in respect to its internal organization, and in relationship to each of the other five dimensions of the model. Change within one dimension will impact other dimensions in an ever-unfolding diffusion of responses to change. Change can be externally driven or inherent to the dynamic nature of any of the six dimensions.

**Fig. 2:**
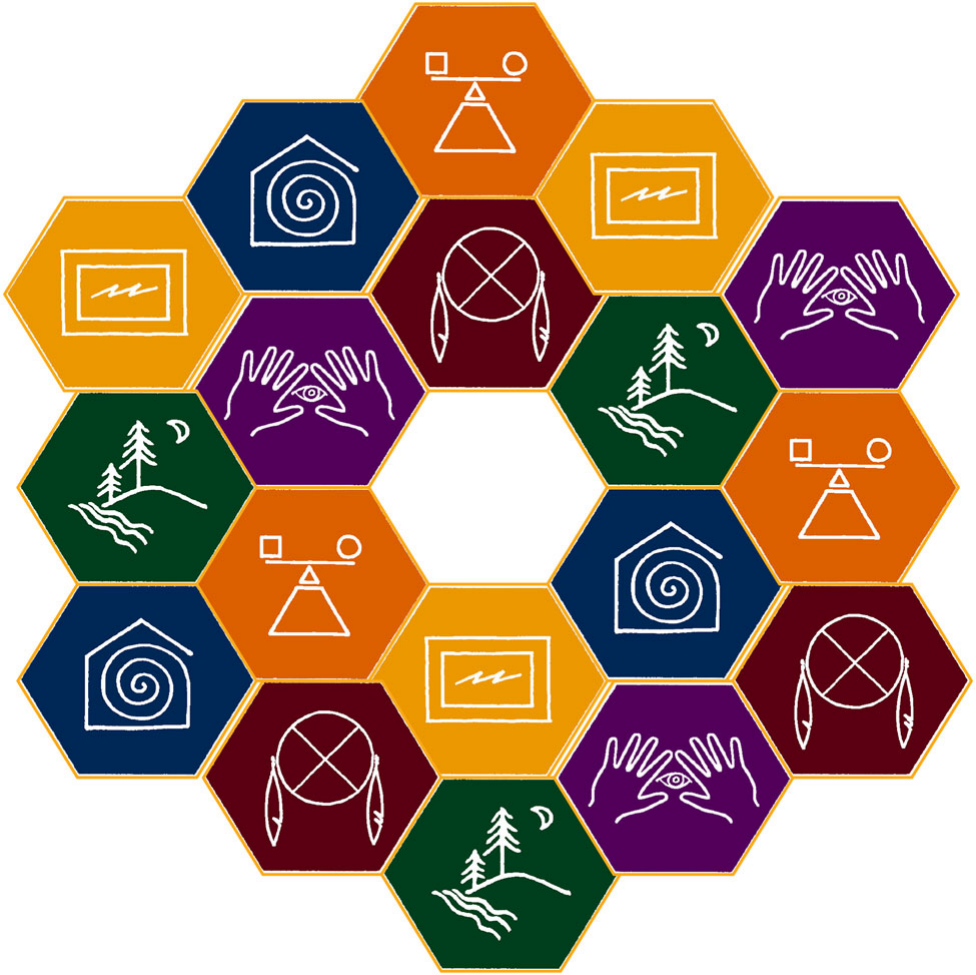
Illustration of tensions among dimensions in the College of Menominee Nation Sustainable Development Institute’s Model. Tensions are represented anywhere the model dimension’s borders touch. An unlimited number of tensions can be illustrated by placing dimensions adjacent to each other (CMN c. 1999)

The SDI model recognizes that there will always be tensions within and among model dimensions. Tensions can be illustrated by placing SDI model dimensions adjacent to one another (Fig. 2). Furthermore, as tensions among model dimensions are relieved new tensions will arise. Because new tensions will always arise, sustainable development is defined as a continual, and sometimes iterative, process.

There are many ways to use the SDI model but it is important to identify the relationships among different dimensions, identify the tensions, and seek solutions to relieve those tensions. For example, Eastern white pine (*Pinus strobus*) and aspen (*Populus tremuloides*) are common tree species in the Menominee forest and require disturbance to regenerate (natural environment). Foresters regenerate these species by using clearcutting and other silvicultural techniques to mimic natural disturbance (technology). Some community members may disagree with clearcutting because they perceive (human perception activity and behavior) that it damages the Menominee forest ecosystem, the community, and Menominee sovereignty (natural environment and land and sovereignty). On the other hand, Menominee foresters may perceive that without this type of forest management some tree species will not regenerate, old trees will not be replaced by young trees, and the forest will become less diverse (human perception, activity, and behavior; natural environment; and technology). In this example, there are tensions among human perception, technology, and the natural environment dimensions. Once the SDI model is used to identify these tensions, it can be used to develop potential solutions. One possible solution is that the Menominee school system (institution) could teach students more about the ecological requirements of certain tree species (natural environment) and about forest management (technology) which could change community perceptions (human perception, activity, and behavior). Another potential solution could be a change in technology that would allow foresters to develop new harvesting and regeneration techniques (technology) that would be perceived more positively by the Menominee community members who view clearcutting as negative (human perception, activity and behavior). In this example, the SDI model is used to characterize a complex sustainability problem and to develop possible solutions.

The final concept in the SDI model addresses how the Menominee People have balanced the six dimensions of sustainable development over time. To do this the SDI model identifies Menominee autochthony as a core cultural value and key component of the SDI model. The Menominee people say that they have always resided in the area of what is now called Northeast Wisconsin, the Upper Peninsula of Michigan, and parts of Illinois; Menominee oral tradition does not include a migration story. Menominee oral tradition says the origins of their people and their “early social and civic governmental organization” came from the mouth of the Menominee River (Grignon et al. 1998). The SDI model postulates that Menominee autochthonic beliefs—the belief that their people come from the land where they live—have allowed them to balance the tensions between the six model dimensions through time. This cultural value has allowed the Menominee to build a strong community, maintain their forest, develop and implement sustainable forestry management practices, and operate a sawmill since the mid-1800s.

The term autochthony (often in contrast to the term indigeneity) has been debated within anthropology, international development studies, and American Indian Studies over the years (see for example Kuper 2003; Asch et al. 2004, 2006; Ceuppens and Geschiere 2005; Dove 2006; Geschiere and Jackson 2006; Pelican 2009; Gausset et al. 2011; Cameron 2012; Greenberg and Greenberg 2013; Keitumetse 2013). Autochthony means coming from the land or soil. Debates arise from different interpretations of what coming from the land or soil means. In one sense it means local or from one area. This interpretation has the potential to marginalize groups as only being knowledgeable about one small or localized area. In the global debates about climate change, for example, this could suggest that local communities would not have much to offer the broader global debate beyond their geographical limits (see Cameron 2012). Other scholars explain that being autochthonous implies a territorial claim (Geschiere and Jackson 2006). Violent struggles for land and power in Africa have been attributed to the use of the term and to the practical implications of using the concept of autochthony to imply that one group belongs in a place or country because they have been there longer while other groups do not (Geschiere and Jackson 2006; Pelican 2009).

The College of Menominee Nation’s use of this term evolved outside of these academic debates and discussion on this topic. The term autochthony is used in the SDI model to describe the belief that the Menominee people originated from the land near where they currently reside. The term also describes the Menominee people’s profound sense of place and their intimate relationship with place. The SDI model does not use the term autochthony to describe a territorial claim or to control the land; territorial control and decision making authority is covered within the land and sovereignty dimension of the SDI model. Autochthony in the SDI model implies a cultural value and belief that the health of the land and people are one—a profound connection and relationship with the land. The fact the Menominee people believe that they were created from the land helps them balance decisions for sustainability. The SDI model recognizes that their tie to the land is not exclusive and that many communities—especially other indigenous communities—share similar values and connections to place. The SDI model encourages groups to understand their own cultural beliefs and values that could be used to balance the six SDI model dimensions. Alternatively, other communities could substitute “sense of place” as a value used to balance SDI model dimensions. Finally, the dynamic nature of the SDI model and its applicability to multiple scales requires recognition that all people and communities are connected.

## Using the SDI model in higher education

During the past decade universities throughout the world have been engaged in developing sustainability science as an interdisciplinary discipline and to teach students about sustainability and sustainable development (Tamura and Uegaki 2012; Yarime et al. 2012). While there is a value in educating students to be interdisciplinary problem-solvers (Johnston and Johnston 2013), only recently have universities begun to integrate sustainability science throughout the curriculum (Cortese and Hattan 2010; Wiek et al. 2011). Furthermore, there has been little research documenting sustainability education at Tribal Colleges and Universities. The following section of the paper will briefly outline two examples of how the SDI model has been used in undergraduate courses at CMN.

### SDI model and sustainable development 100: introduction to sustainable development

Sustainable development has been a core value at the CMN since its founding in 1993. From the beginning there have been formal course offerings and an academic major in sustainable development. In an effort to bring sustainability concepts into every academic major, in 2005 the CMN administration required all students to take Sustainable Development 100: Introduction to Sustainable Development as requirement for graduation. To date, hundreds of CMN students have taken the course and have applied the principles and values of sustainability to their careers and lifestyles. The foundation of the course lies in the SDI model and the Menominee sustainable forestry tradition. Sustainability knowledge is applied to develop broader academic skills and competencies that encourage the students to become actors in a complex world at local and global levels. Students learn to question the current dominant forms of knowledge that have contributed to our unsustainable world and engage them in thinking about solutions to these environmental and social sustainability challenges. This type of critical thinking is important to integrate sustainability in higher education (American College Personnel Association 2008).

The SDI model is used as a framework for the students to explore the breadth and depth of sustainable development by thinking about each of the six dimensions, their interactions, and how those have changed through time. Students learn that sustainability entails complex interactions among topics such as climate change, biodiversity, social justice, technology, food security, population control, land rights, organizational development, cultural preservation, poverty alleviation, economic development, resource management, and spirituality. When students begin to look at the six dimensions of the SDI model they begin to understand that sustainability involves all aspects of life and their coursework.

Barlett and Chase (2013) argue that “the endpoint of the multiple paths to sustainability is elusive because of the dynamic, evolving, and transient nature of our understandings of both the challenges and the solutions, both locally and around the world today.” The introductory class at the College of Menominee Nation does not cover all aspects of the depth and richness of sustainability; however, it shows each student, no matter what their academic or career goals might be, that they are an integral part of a sustainable future. Each dimension can adversely affect other SDI model dimensions. On the other hand, each dimension also can positively affect other SDI model dimensions. Sustainable development, defined by the SDI model, gives as much attention to finding solutions as it does to diagnosing problems. Therefore, to affect positive change the SDI model helps students think critically about how change in one dimension could result in a positive rippling effect on the other dimensions.

The Global Perspectives Inventory (GPI, see https://gpi.central.edu/) is used by CMN to assess student learning and their understanding of multiple cultural perspectives, holistic development, and global connections. In one study, students were given the GPI test before and after taking Introduction to Sustainable Development. Student results on the pre-test were similar to the results of freshman and sophomores from other universities. After taking the course, student test results increased to levels equivalent to college seniors (Van Lopik 2013a). Because of this study and its results, all CMN students are given the GPI test and they consistently score higher than national averages. CMN attributes these results in large part to the Introduction to Sustainable Development course where the SDI model is used as a framework. Furthermore, students consistently indicate through end of semester course evaluations that the course is relevant to their lives, other courses, and their career paths. They also indicate that the SDI model was useful to them in understanding the complex and interconnected nature of sustainability. One student summarized a typical response by stating, “this class has really opened my eyes to what goes on in the world and what needs to be done to keep our Earth healthy” (Van Lopik 2013b).

### SDI model and anthropology 200: introduction to Native American cultures

While it is important, if not crucial, to have an academic home for sustainable development and sustainability sciences curricula (Wiek et al. 2011; Yarime et al. 2012), there are also opportunities to integrate the core components of sustainability into college courses within established disciplines (Barth and Michelsen 2013). An example of this occurs in Anthropology 200: Introduction to Native American Cultures. The course rests on a dual framework, drawn equally from anthropology and from the SDI model. The course explicitly incorporates the SDI model from an anthropological perspective which reinforces student learning of anthropological concepts such as holism, participant observation, cultural relativism, and transcultural realities. While it is important to acknowledge the origin of the SDI model in the Menominee forest management tradition, its utility to anthropology is its breadth, universality, holistic perspective, and the power of its central “autochthony”. Basic anthropology concepts are similar to concepts in the sustainability sciences (Croll and Parkin 1992) and the SDI model integrates the two.

The Introduction to Native American Cultures course focuses on the universality of the SDI model and uses the framework to analyze culture. Source material for the course include: students’ own cultures and experiences, printed matter (especially books written by Native American authors), films and other visual sources, and cultural observations using the anthropological method of participant observation. While the sources vary, the SDI model provides a framework that intertwines with anthropological concepts in course discussions, daily topics, and assignments. Integration of anthropology and sustainability science is achieved by introducing students to the SDI model on the first day of class and continuing to focus on one or more of the six dimensions during each subsequent class meeting. Furthermore, reading assignments and responses to films begin by analyzing one, then two, and then up to all six dimensions of the SDI model which provides a transition to complex interdisciplinary analysis by the end of the semester.

Results from student papers, evaluations, and tests indicate students can integrate the SDI model into anthropology. Testing shows that most students can apply the six SDI model dimensions across cultures. Additionally, based on classroom discussions and course assignments, many students become aware of the interactions among and the interconnectedness of the six SDI model dimensions by the end of the semester. Furthermore, students often begin to apply the SDI model to what they are learning in other courses and share those perceptions and insights in other classes. This spreads sustainability science perspectives further across the CMN curriculum. Students also begin to use the SDI model to make sustainability a feature with which to analyze cultural patterns across multiple cultures including their own cultures. Students have also shared their insights with their community and nationally as evidenced by two articles published by a student based on his ethnographic paper about the role of culture and language in Menominee community revitalization (Arthur 2013a, b). In these ways, the SDI model fosters a holistic perspective where students are able to integrate culture and sustainability.

The Introduction to Sustainable Development and Introduction to Native American Cultures courses show how the SDI model have be used to integrate sustainability science across the curriculum and culture at CMN. While there are some features in common across Native American cultures, such as concentric circles, no two cultural systems are identical (see Cajete 1999; Cornelius 1999). The general flexibility of the SDI model dimensions are reflective of life as experienced and understood by Native Americans. The six dynamic dimensions and interactions of the SDI model function across cultural contexts and provide students opportunities to realize how their own life experiences and indigenous perspectives are represented in a holistic integration of culture and sustainability science.

## Using the SDI model for participatory community planning and research

Scientists, academics, and indigenous communities are all looking for methodologies to foster interdisciplinary research and community participatory planning that incorporates traditional ecological knowledge, community perspectives, and multiple scientific disciplines (Huntington 2000; Kauffman 2009; Schoolman et al. 2012). The SDI model can be used as a framework to organize, conduct, and analyze transdisciplinary projects which incorporate multiple scientific disciplines along with traditional ways of knowing. The SDI model can be used to develop a narrative by framing questions and answers around each model element and each interaction (Table 2). The narrative can take many forms. It can be written, spoken, or even drawn by participants. The narrative can incorporate quantitative and qualitative data. The remainder of the paper will outline two examples of how the SDI model can be used in community planning and participatory research. The first example describes how the SDI model has been shared with indigenous communities and development workers in Bolivia. The second example describes how the SDI model was used to develop a framework to understand community capacity to respond and adapt to changing climates in the Great Lakes Region of the United States.

**Table 2:** The Sustainable Development Institute Model can be used by communities, planners, educators, and researchers to create a complex narrative to understand the past and present and create visions and solutions for the future

SDI model dimension	Possible questions to develop narrative
Land and sovereignty	Does the community have control over their resources? In the past? In the future? Who makes decisions? How does this affect the other model elements?
Natural environment	How has the natural environment changed over time? How do people interact with the natural environment? In the past? In the future? How do these changes affect institutions, perceptions…?
Institutions	How are community institutions organized? In the past? In the future? How have institutions changed over time? How do the institutions affect human perceptions, natural environment…?
Technology	How is technology used to influence natural environment, perceptions, institutions? In the past? In the future? How is technology used in a community? How has technology changed over time?
Economy	How does the local economy work? In the past? In the future? How does the global economy influence the local economy? How does the economy affect the other model elements?
Human perception, activity, and behavior	How do individuals perceive forest management? The community? How have perceptions and behaviors changed over time? How does this affect institutions, natural environment…?
Menominee autochthony (profound sense of place/tie to the land)	How does the community perceive their sense of place? What values frame community decisions? How does sense of place affect other model elements? What are other community values that influence decisions?

The table lists some questions that can be used for creating the narrative

### SDI model and sustainable development in Bolivia

The SDI model was shared with several indigenous communities, indigenous organizations, and sustainable forestry organizations in Bolivia during research and technical assistance trips in 2008 and 2009. During these meetings, the history of Menominee forest management was presented along with a basic explanation of the SDI theoretical model and its dimensions. The SDI model was then used to discuss several local examples to demonstrate how the SDI model could be used in constructing narratives about sustainability. Additionally, it was explained that the Menominee have been able to balance tensions among the six elements because of their belief that they come from the land close to their current reservation. This was explained to be an important Menominee cultural value.

Communities using the SDI model have to identify core community values that drive decision making and that could be used to understand and balance the tensions among the six model dimensions. It was explained that indigenous communities may think of this as a key cultural value like, water is sacred or that the health of a cultural keystone species is important. Some of the Bolivian groups responded by saying that they needed to go and think about their traditional stories to find their cultural values that would help them mediate the tensions among the dimensions. Other groups asked that presentations be given to broader audiences within the community to start discussions. Most groups also wanted to discuss the relationship between land and sovereignty, what that means in the United States and what it could mean for their communities with their own unique histories, cultures, and laws.

The positive reaction from presenting the model in Bolivia suggested the universal nature of the SDI model. It also highlighted the need to incorporate complexity and indigenous values into sustainable development projects and planning. Finally, it was important for indigenous communities to hear about the Menominee experience and to learn that it was possible to manage resources sustainably and use forestry to exercise control over their territories, that indigenous people in the USA have organized their own colleges and universities, and that those institutions were able to develop and share a complex model of sustainable development. Sharing the SDI model and the Menominee experience can bring hope to other communities engaged in incorporating their cultural values into sustainable natural resource management, planning, and development (see Bernard and Young 1997; Bernard 2010).

### SDI model for participatory research

In 2012 CMN received funding from the Great Lakes Integrated Sciences and Assessments Program for a project titled “Supporting Tribal Climate Change Adaptation Planning through Community Participatory Strategic Foresight Scenario Development”. The goal of the project was to provide tribal communities and tribal natural resource management departments with information on climate science, traditional ecological knowledge, and community input to be used to develop a range of possible future scenarios for each tribal community. The scenarios were designed to be useful in developing climate adaptation plans that incorporated specific concerns for each tribal community.

Developing scenarios typically involves identifying trends, drivers of change, and fundamental uncertainties. Scenarios can be developed around themes like: a business as usual scenario, an economic collapse scenario, an ecological change scenario, and a positive transformational scenario (Dator 1979, 2002; Schwartz 1996; Amer et al. 2013). One difficulty in developing scenarios that are useful for climate change adaptation planning is to ensure that there are a broad range of drivers to think about for each scenario; scenarios are more insightful with broader perspectives going into their development (Peterson et al. 2003). The SDI model provides a framework for identifying a broad range of drivers and was used by the project team and tribal community partners to identify system drivers and to stimulate in-depth interdisciplinary discussions around the potential future impacts of climate change. To do this, an initial matrix of system drivers was developed by the project team based on the SDI model dimensions. Each of the tribal communities identified the variables important for their communities under each SDI model dimension and used them to explore multiple future scenarios.

Using the SDI model as a framework to develop this participatory research and planning project ensured that participants discussed a broad range of system drivers and scenarios. For example, climate change impacts on tribal governance is generally not a topic discussed during climate change meetings organized by physical or biological scientists. The SDI model framework ensured that those discussions occurred because participants needed to address each of the six model dimensions, including the land and sovereignty and institution dimensions. The SDI model ensured that each scenario was discussed holistically by forcing participants to address system drivers and the relationships among drivers for each SDI model dimension. Finally, once the narrative scenarios were developed and system drivers identified, the discussion moved to identify the cultural values that grounded each community and could be used to balance tensions and craft sustainable solutions. Participants discussed the question, “what cultural value would allow your community balance and resolve tensions among each system driver (SDI model dimension), what capacities does your community have, and what capacities are needed to address issues in each scenario?”

In both the Bolivian and American Indian examples, the SDI model proved to be a valuable framework to discuss sustainability, integrate multiple perspectives, and incorporate indigenous and community values. The SDI model afforded each community a structured framework with which to engage in participatory research and community planning processes.

## Conclusion

This paper brings an indigenous perspective to discussions defining sustainability and adds to the literature by outlining a novel theoretical framework with which to understand sustainability. In some ways the SDI model reflects current understandings of sustainability science; sustainability is iterative, it is a process, it includes multiple perspectives and multiple disciplines. In other ways the SDI model reflects the values and the lived experience of the Menominee people where cultural understandings of the interconnectedness of essential dimensions of sustainability can be applied to sustainability science in many situations. The SDI model has provided students, researchers, and community members with a framework to holistically engage with complex issues of sustainability science and to build sustainable solutions. The SDI model has proven to provide a theoretical framework with which to understand sustainability within the context of indigenous values and perspectives. This paper outlined several examples of how the SDI model has been successful in higher education, community planning, and participatory research. We argued that a narrative based on the SDI model can incorporate history, complex interactions, culture and change. One of the greatest strengths of the SDI model is its usefulness in outlining universal dimensions of sustainability appealing to indigenous and non-indigenous people alike. However, because it was developed at an indigenous institution for higher education it easily incorporates indigenous values into sustainability discussions—something that communities, scholars, and practitioners have been struggling to do for decades. We intend this paper to be a starting point for others to begin to incorporate the SDI model and its concepts into their own projects and to encourage a larger discussion about the components of sustainability and how best to incorporate them into sustainability science and sustainable development.
